# Recipes

**Published:** 1871-01

**Authors:** 


### RECIPES.

CLEANING SILVER PLATED WARE.--The use of soap gives them a leaden hue. When tarnished, rub them with a little fine whiting wet with water; then wash them clean in soft, warm water. Dry them, after which polish with fine whiting on a soft chamois skin.

---

INDELIBLE INK.—Dissolve four parts of aniline black in 16 parts by weight, of alcohol, with 60 drops of strong hydrochloric acid, and dilute the dark blue solution with 90 parts by weight, of water, in which six parts of gum arabic has been previously dissolved. This ink is said not to act upon steel pens or to suffer any alteration by alkalies or acids.

---

TO GET RID OF ROACHES.—Sprinkle powdered borax in the places where they congregate. This is one of the cleanest and safest means of putting an end to their visits. A friend, one who knows, says dry "paris green" paint, sprinkled in their haunts will surely drive them away. In using the paint do not place it where it is liable to get among food, as it is highly poisonous.

WASHING FLUID.—The following fluid is a valuable one for assisting in large washings. It renders the clothes white and clean with less than half the rubbing, and does not injure them: Dissolve 1¼ lbs. of washing soda and ¼ lb. of borax in a gallon of water, by boiling. When the solution is cold, add a half tea-cupfull of aqua ammonia (spirits of hartshorn), and then place the whole in a well corked jug. Use a tea-cupfull to every pail of water, in washing.

TO CLEAN GLOVES.—The ladies will be glad to learn how to make their old kid gloves almost as good as new. Make a thick mucilage by boiling a handful of flaxseed; add a little dissolved soap; then when the mixture cools, place the gloves on the hands, and with a piece of white flannel, rub the mixture upon the gloves until they are clean. Use only enough of the mixture to remove the dirt without wetting entirely through the glove. Then rub them dry before a fire.

